# Nanobiotechnology and blood substitutes

**DOI:** 10.4103/0973-6247.76004

**Published:** 2011-01

**Authors:** Rajiv Saini, Santosh Saini, Sugandha Sharma

**Affiliations:** Department of Periodontology and Oral Implantology, Rural Dental College-Loni, Maharashtra, India; 1 Department of Microbiology, Rural Dental College-Loni, Maharashtra, India; 2 Department of Prosthodontics, Rural Dental College-Loni, Maharashtra, India

Sir,

Nanobiotechnology is the assembling of biological molecules into 1–100 nm dimensions. These dimensions can be the diameter of nanodimension artificial cells or particles; membranes with nanodimension thickness or nanotubules with nanodimension diameter.[1] Since red blood cell (RBC) membrane includes blood group antigens, typing and matching are required earlier than they can be transfused into patients; these result in delays in emergency situations. The storage time using regular method is merely about 42 days. RBCs cannot be sterilized to remove infective agents like hepatitis viruses, HIV, and other probable emerging infective agents. Thus, RBCs substitutes are being developed. RBC contains hemoglobin (Hb), antioxidant enzymes, and multienzyme system to prevent the conversion of Hb into nonfunctioning met Hb. It has been shown as far back as 1957 that artificial RBC can be prepared with ultrathin polymer membranes of nanodimension thickness. To increase the circulation time, the first-generation engineered Hb is formed by using glutaraldehyde to crosslink Hb into soluble nanodimension polyhemoglobin (poly-Hb) that has been tested clinically in patients. Further extension includes conjugated Hb, intramolecularly crosslinked Hb, and recombinant Hb. For certain clinical uses, in addition to engineered Hb, antioxidants need to remove oxygen radicals to prevent injury from ischemia reperfusion. Nanobiotechnology is used to prepare second-generation engineered Hb by assembling Hb together with superoxide dismutase (SOD) and catalase (CAT) to form a nanodimension soluble complex of poly-Hb-CAT-SOD. A third-generation system is to prepare nanodimension complete artificial RBCs that can circulate for sufficient length of time after infusion. One approach uses lipid vesicles to encapsulate Hb. Another approach to use biodegradable polymer-like polylactic acid or a copolymer of polyethylene glycol–polylactide (PEG-PLA) to form the membrane of nanodimension completes artificial RBC.[2] Past experience has shown that it takes many years to develop ideas on blood substitutes into products and that lack of basic information has resulted in much failure and delays. It is important to carry out basic research to gain important basic information needed for the simultaneous development of blood substitutes. In the meantime, two types of first-generation nanodimension poly-Hb are in the final stages of clinical trials in human and one of these has been approved for routine clinical uses in patients in South Africa. New nanodimension-conjugated Hb is also being tested in clinical trial. Shortage of human Hb is being resolved by studies on recombinant human Hb, placenta Hb, bovine Hb, and synthetic heme. Meanwhile, new generations of modified Hb are being developed that can modulate the effects of nitric oxide for those clinical applications that might have potential problems related to oxygen radicals. Poly-Hb can be crosslinked to an enzyme to suppress the growth of tumor. A further development is the use of PEG-lipids or PEG-biodegradable polymer membranes to prepare nanodimension artificial RBCs containing Hb and complex enzyme systems. Many other extensions and modifications of this general principle of blood transfusion in nanobiotechnology are possible.
